# LANGE/PAROLE, STRUCTURE/ACTION: TOWARD A REFRAMING OF AGENCY IN GERONTOLOGY AND LIFE COURSE STUDIES

**DOI:** 10.1093/geroni/igae098.0177

**Published:** 2024-12-31

**Authors:** Dale Dannefer, Nan Zhou

**Affiliations:** Case Western Reserve University, Cleveland, Ohio, United States; Case Western Reserve University, Cleveland, Ohio, United States

## Abstract

Almost by definition, “Emancipatory Gerontology” carries implications like “choice” and “freedom” which in gerontology have often presupposed what is often termed “agency”. Agency remains a popular concept in gerontology and related fields such as life-course studies when, e.g., exploring such matters as life transitions and decision-making in areas like family formation or retirement. Yet its often-glib and superficial invocation reflects daunting problems identified in recent scholarship, concerning agency’s conceptualization and measurement. For any construct, adequate measurement presupposes a sound conceptual definition. The very definition of agency remains unresolved and problematic, despite numerous recent efforts at theoretical reformulation and refinement. Some issues revolve around nuances in the history of the term and its dimensions, others around issues of measurement. We contend that an even more fundamental set of problems arise because of a continued lack of recognition of the force of culture-specific symbol systems and social structure to frame the categories and nuances of thought within which agentic intentions are externalized in action. For example, no one chooses their native language, yet even freely chosen and passionately pursued “agentic” acts represent an expression of conscious intent formulated within perceptions, aesthetics, etc., structured by that language and its accompanying culture. We suggest that the structure-agency tension corresponds closely to the distinction between language and speech (langue/parole) articulated by de Saussure, and to related insights offered by Bourdieu. We contend that emancipation ultimately requires rigorous attention to constraint, and hence recognition of the structuring of agency by unrecognized and naturalized features of context.

